# Prehabilitation program for African sub-Saharan surgical patients is an unmet need

**DOI:** 10.11604/pamj.2020.36.62.21203

**Published:** 2020-06-03

**Authors:** Antero do Vale Fernandes, Daniel Moreira-Gonçalves, Jotamo Come, Nilton Caetano Rosa, Victor Costa, Lygia Vieira Lopes, Paulo Matos da Costa, Lúcio Lara Santos

**Affiliations:** 1 Experimental Pathology and Therapeutics Group of Portuguese Institute of Oncology of Porto Francisco Gentil, E.P.E (IPO-Porto), Portugal,; 2 Intensive Care Service of Hospital Garcia de Orta, E.P.E, Almada, Portugal,; 3 Research Centre in Physical Activity, Health and Leisure (CIAFEL), Faculty of Sport, University of Porto, Porto, Portugal,; 4 Surgical Department of Maputo Central Hospital, Maputo, Mozambique,; 5 Surgical Oncology Department of Angolan Institute Against Cancer, Luanda, Angola,; 6 Surgical Department of Agostinho Neto Hospital, Praia, Cape Verde,; 7 Oncological Unit of Sagrada Esperança Clinic, Luanda, Angola,; 8 General Surgery Service, Hospital Garcia de Orta, E.P.E, Almada, Portugal,; 9 Faculty of Medicine of the University of Lisbon, Lisbon, Portugal,; 10 Surgical Oncology Department of Portuguese Institute of Oncology of Porto Francisco Gentil, E.P.E (IPO-Porto), Portugal,; 11 ONCOCIR, Education and Care in Oncology, Lusophone Africa, Angola

**Keywords:** Africa, patients, postoperative, surgery, risk

## Abstract

Approximately 4.2 million people worldwide die within 30 days of surgery each year. Half of these deaths occur in low- and middle-income countries. Postoperative deaths account for 7.7% of all deaths globally, making it the third-highest contributor to deaths, after heart disease and stroke. In sub-Saharan Africa, there is a higher rate of mortality following postoperative complications compared to high-income countries. The WHO has tools to help countries provide safer surgery. However, implementation remains poor in most African countries. Interventions focused on intraoperative or postoperative measures to improve perioperative prognosis may be too late for high-risk patients. Poor preoperative cardiorespiratory functional capacity, poor management of pre-existing comorbidities and risk factors and no assessment of the patient´s surgical risk is associated with adverse postoperative outcomes, including mortality, complications, slower recovery, longer intensive care stay, extended hospital length of stay and reduced postoperative quality of life. To significantly decrease morbidity and mortality following surgery in Africa, we propose the implementation of a comprehensive preoperative intervention, that must include: i) risk assessment of surgical patients to identify those at greater risk of postoperative complications for elective surgery; ii) increase the preoperative functional reserve of these high-risk patients, to enhance their tolerance to surgical stress and improve postoperative recovery; iii) anticipate postoperative care needs and organize tools, resources and establish simple workflows to manage postoperative complications. We believe this approach is simple, feasible and will significantly reduce postoperative burden for both patients, hospitals and society.

## Essay

An African continental study of 25 low- and middle-income countries was recently published by Biccard BM *et al*. characterizing perioperative outcomes of 11193 surgical patients. The patients were 66.4% women, 87.3% classified with an American Society of Anesthesiologists (ASA) score of I and II, 29.7% undergoing major surgery and 57.1% urgent/emergency surgery [1]. Arterial hypertension (16.3%), diabetes mellitus (6.8%) and HIV positive/AIDS (11%) were the main comorbidities of the operated patients. Non-communicable diseases (NCD) were the most frequent indication for surgical treatment (42.2%), followed by caesarean section (27.3%), trauma (17.8%) and acute infection (12.7%). Despite being younger, presenting a lower risk profile and low complication rates, surgical patients in Africa were twice as likely to die after surgery in comparison to the global average. Indeed, in hospital mortality was 2.1%, with 18% developing postoperative complications (POC) and 9.5% of the patients died following POC [1]. When considering elective surgery (ES) only, mortality occurred in 1% of 4658 patients, with an incidence of postoperative complications of 13.4% and death after POC of 4.8%.

A greater incidence of POC and death were reported following surgery for NCD (37.3% and 40.3%, respectively), infection (20.2% and 26.5%, respectively) and trauma (20.5% and 25.5%, respectively). Infectious, cardiovascular and respiratory complications were the most prevalent [1]. The factors contributing to POC and death are multifactorial and may include insufficient medical staff, poor infrastructure, low procedural volumes and failure to identify and/or treat POC by health professionals [2,3]. Intensive care admission should also be scheduled in advance. In their study, Biccard BM *et al*. [1] identified that only 16.3% of patients who developed POC, the vast majority after ES, were admitted to intensive care units (ICU) to prevent and treat early complications. The lack of immediate postoperative surveillance and intervention is responsible for many deaths in many African countries. Therefore, acute care surgery (ACS) services should be implemented even in a low-resource setting [4].

**Figure 1:**
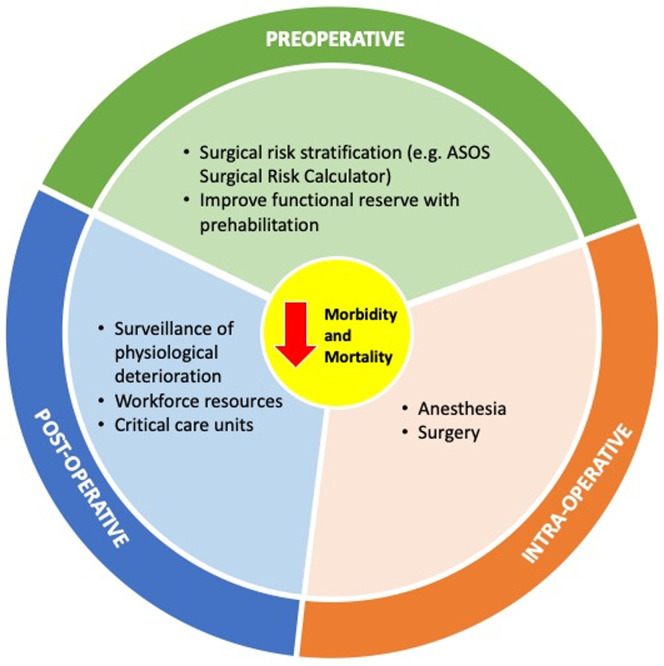
stratification of measures to decrease perioperative morbidity and mortality in surgical patients

In Rwanda, the implementation of an ACS service resulted in decreased length of hospital stay [5]. Thus, while surgical care is a major need for African countries, surgical outcomes will remain poor unless effective perioperative care based on affordable resources is made universally available. Perioperative care is a multicomponent intervention implemented by a multidisciplinary team with the purpose to provide safe surgery, accelerate recovery and reduce morbidity and mortality (Figure 1). While the intra- and postoperative care have already received some attention, the potential of the perioperative period remains poorly explored in African countries [6-11]. This time frame represents a major opportunity for decreasing postoperative morbidity and mortality through appropriate surgical risk stratification and patient optimization [12].

**Risk assessment of surgical complications in sub-Saharan Africa:** in an environment with limited resources for postoperative care, the early identification of high-risk patients for POC is likely to be a key factor to consider. Several tools are available to estimate perioperative risk for both planned and emergency surgeries in high-income countries [13,14]. However, their use in low-income countries is often limited because the pattern of risk for poor outcomes differs from high-income countries and due to the lack of resources, the access to biochemical and imagological tests required by more sophisticated tools is reduced [15]. Recently, Kluyts H-L *et al*. proposed the use of the ASOS surgical risk calculator as a simple tool to identify African surgical patients at risk for in-hospital postoperative mortality and severe complications and thus, to identify those patients in greater need for enhanced postoperative surveillance [16]. However, its external validation needs to be assessed before. To predict complications and risk of death before surgery, other African authors have conducted relevant studies, including the use of online tools, provided that this tool has wide distribution [17,18]. This is a field that deserves further research effort as it may greatly contribute to save lives.

**Estimating the risk of complications and mortality of surgical patients before surgery can be helpful:** risk stratification of patients is supposed to support better decisions by informing about the risks and benefits of proceeding with surgery, about discussing treatment alternatives and guide the use of available resources, with the ultimate purpose of improving postoperative outcomes. Ntobeko Ntusi, a South African cardiologist, in a recent editorial in the South African Medical Journal, asked: “does the preoperative evaluation of patients improves surgical outcomes?”. He found that the data on the effect of preoperative medical consultation on cost measures is conflicting [19]. While some studies reveal a decrease in-hospital stay after preoperative evaluation and care of patients [20,21], other studies have shown an increase in costs and a similar length of stay for consulted patients [22-24]. He also points that while medical teams can successfully identify conditions that may affect surgical outcomes, it is not clear if they make evidence-based recommendations to target those conditions and assuming they make it, it is also not clear if the consultative recommendations are implemented [19]. With this data and his experience, Ntusi argued that an experienced perioperative medicine physician should be able to identify the pertinent medical problems, anticipate potential perioperative problems, recommend evidence-based interventions to optimize the patient and communicate and work effectively with all the preoperative team members (e.g. nursing, physiotherapist, medical, surgical and anesthetic) [19]. Thus, to deal with the problem of postoperative morbidity and mortality, the perioperative care, particularly the potential of the pre-surgical period to optimize the patient for surgery, needs to be taken more seriously. These patients need and deserve better care and prehabilitation programs can make the difference once incorporated in the routine practice of surgical teams.

**Prehabilitation to prepare for surgery:** the impact of surgery leads to significant homeostatic disturbance which, together with reduced functional capacity (physical, nutritional and psychological status) and poor medical optimization (e.g. unappropriated management of chronic diseases, anemia, hypertension, hyperglycemia and smoking), act as risk factors for negative surgical outcomes [25]. Prehabilitation is a multimodal strategy implemented in the preoperative period, aiming to increase preoperative functional reserve and leading to better postoperative functional recovery and reduced incidence of complications. In practice, prehabilitation programs may include cardiovascular and resistance training exercises, nutritional advice designed to support an increase in lean body mass, the introduction of coping strategies to deal with surgical anxiety, smoke cessation support, treating preoperative anemia and other modifiable risk factors [26]. An increasing number of studies support the safety, feasibility and efficacy of multimodal prehabilitation to improve surgical outcomes in cancer patients undergoing major abdominal and cardiothoracic surgery [27]. The benefits range from lower rate of postoperative complications, to less deterioration of physical function and better quality of life [28]. However, this evidence comes mainly from high-income countries and thus, there is an urgent need to test the potential of prehabilitation programs in African countries.

## Conclusion

Decreasing morbidity and mortality following surgery in Africa will require adequate perioperative optimization and better postoperative care planning. Preoperative diagnosis of comorbidities and social habits that are considered risk conditions for surgery should be identified throughout appropriate risk assessment tools. Patients considered to be at high-risk for complications following surgery should be proposed for prehabilitation to increase their preoperative functional reserve and enhance recovery following surgical treatment. The knowledge about most common surgical complications should be used to anticipate postoperative burden, care needs and organize available resources in advance. We believe that this approach to perioperative care will play a decisive role in sub-Saharan Africa in changing surgical morbidity and mortality for better.
